# Quality Improvement Project to Evaluate Discharge Prescriptions in Children With Cystic Fibrosis

**DOI:** 10.1097/pq9.0000000000000208

**Published:** 2019-09-06

**Authors:** Matilde Merino Sanjuán, Veronica Chorro-Mari, Chinedu Nwokoro, Nanna Christiansen, Caroline Pao, David Gomez-Pastrana Duran, Monica Climente Marti

**Affiliations:** From the * Catedrática de Universidad, Farmacia y Tecnología Farmacéutica, Facultad de Farmacia, Universidad de Valencia, Burjassot, Valencia, Spain; † Paediatric Pharmacy Department, Barts Health NHS Trust, London, United Kingdom; ‡ Paediatric Respiratory Department, Barts Health NHS Trust, London, United Kingdom; § Pharmaceutical Technology Department, Valencia University, Spain.

## Abstract

Cystic Fibrosis (CF) requires multiple pharmaceutical treatments, elevating the risk of medication errors (ME), which may compromise patient safety. This study aimed to improve the quality of discharge prescriptions (DPs) using indicators following admissions for IV antibiotics in pediatric CF patients.

**Methods:**

This project involved a longitudinal observational retrospective descriptive study followed by a longitudinal quasi-experimental prospective phase between January 2013 and December 2016 in CF patients admitted to a London Children’s Hospital. The CF pharmacist reviewed DPs. Six rights of medication administration were defined (6R): dose, drug, frequency, duration of treatment, pharmaceutical form, and route of administration. We classified ME according to 6R, including subtype of error: committed/omitted. We calculated quality indicators by dividing the number of each correct parameter defined by 6R by number of DPs. Retrospective results were used prospectively to describe and implement improvement strategies and safety actions.

**Results:**

The retrospective study phase included 42 CF children (100 hospital admissions and 1,343 drugs). The prospective phase included thirty-five children (55 admissions and 822 drugs). The total number of ME identified was 148 (78 committed; 70 omitted) in retrospective phase and 135 (19 committed; 116 omitted) in prospective phase. Quality indicators for drug and dose showed significant improvement after implementing safety strategies. The global quality indicator increased from 22% (retrospective) to 41.82% (prospective), but we did not achieve the previously defined quality standard value (50%).

**Conclusions:**

A retrospective review of DP by a CF Pharmacist identified failures in DP quality. Implementing improvement strategies improved prescribing. Integrating pharmacist within multidisciplinary team improves DP reducing errors.

## INTRODUCTION

Cystic fibrosis (CF) patients have complex medication regimens and undergo frequent hospital admissions. Each of which is an opportunity for medication errors (ME). On admission, the hospital pharmacist performs *medication reconciliation* to clarify and document drug histories. During this process, we identified ME in previous discharge prescriptions (DPs), with resultant confusion regarding the appropriate treatment regimen. This finding necessitated reoptimization of medication at admission to ensure that no harm had occurred as a consequence. Electronic prescribing reduces ME,^1^ and ours lack a computerized order entry system. Therefore, medications need to be typed for dose regimes.

Errors are perhaps inevitable, but prompt and effective identification (with subsequent correction) of contributory factors (a learning culture) will prevent their repetition.^2,3^ In the pediatric CF context, with the combination of frequently adjusted polypharmacy (for disease evolution, changes to treatment guidelines, or simply due to physical growth or maturity) and multiple hospital admissions, this process of learning from errors is particularly pertinent. Formulation information is essential in pediatrics, liquid forms with differing concentrations are widely used, and parents tend to recall liquid volumes rather than actual milligrams or microgram doses, risking 10-fold, or greater dosing errors.

Additionally, inadvertent dispensing of alternate brands may risk both compliance and efficacy, as equivalent strength products from different sources may have markedly differing palatability and even physical properties. Additional risks stem from the frequent use of pediatric formulations, as well as off-label or unlicensed medications, compounding errors, and the fact that junior medical staff does most of the prescribing, many of whom work only briefly within the CF service. The risk of ME in pediatric CF is, therefore, particularly high.

There is increasing evidence that the involvement of the pharmacists in interdisciplinary teams has a positive influence on the quality of medication use and patient safety by rationalizing the pharmacotherapy and reducing ME and drug-related problems in these settings.^4–12^ Furthermore, proactive participation of pharmacist conducting medication reconciliation at admission and discharge is useful to identify, prevent, and resolve drug-related problems.^13^

Previous work has examined ME in different patient populations,^2^ but few have involved CF patients.^14,15^

The main objective of this study was to improve the quality of the DP for CF patients admitted to the hospital to receive IV antibiotics, using safety indicators to drive this improvement.

## METHODS

We performed this study in a specialist pediatric CF center in a London children’s hospital. Improvement actions followed an initial retrospective longitudinal observational descriptive study of 24 months. We implemented safety strategies in the DP process, including a prospective longitudinal quasi-experimental study of 8 months. Included case notes came from subjects 0–16 years of age with a diagnosis of CF who had been admitted to the pediatric respiratory ward to receive intravenous therapy. The Clinical Effectiveness Unit sponsored the study at the host hospital; the study was viewed as a service evaluation and quality improvement with no direct patient impact and thus did not come under ethics review board jurisdiction.

**Fig. 1.**
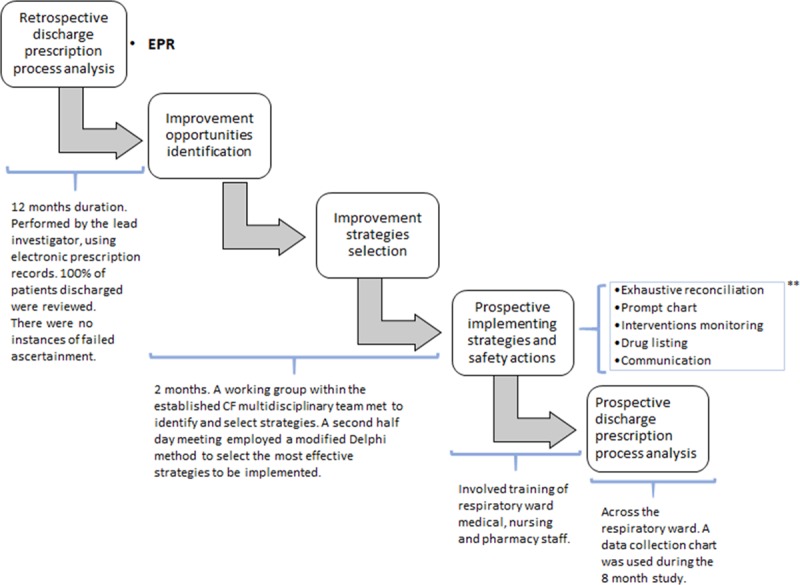
Flowchart process identifying improvements opportunities, selecting and implementing strategies and safety actions, and sources of information used to evaluate therapies prescribed at hospital discharge. ** *Exhaustive reconciliation:* of drug histories using patient’s drugs, homecare prescription, primary care prescription, and previous discharge letter or last clinic letter. *Prompt chart:* designed and attached in drug chart with emphasis on inhaled nebulizers to be reviewed at discharge time. *Interventions monitoring*: during a hospital stay. *Drug listing:* at discharge pharmacist could help to list the drugs that would need to be continued. *Communication:* with careers of any changes, nurses, and prescribers. EPR indicates electronic prescribing in retrospective phase; EPP, electronic prescribing in prospective phase.

Figure 1 depicts how we conducted the study; bold text indicates sources used to detect DPs, opportunities for improvement, and target-optimized prescribing.

We reviewed DPs for the following factors:

1. The presence of the weight of the child in the prescription.
2. The presence of any clinically indicated medicines.
3. Pharmacokinetic aspects (drug interactions, dose adjustment for CF/other drugs/pathology).
4. Optimized dose and frequency for age, anthropometry, hepatic impairment, etc.
5. Treatment duration, start, stop, and efficacy/safety review dates.
6. Route of administration (oral, IV, nebulized, rectal, etc.).
7. Pharmaceutical formulation and specific brand where necessary.
8. Allergy information documented.

We assessed the quality of the DPs according to the 6 Rights (6R) of medication administration: drug, dose, frequency, duration of treatment, route of administration, and pharmaceutical form. We calculated these quality Indicators by quantifying the number of prescriptions with correct completion for each of the 6R and then dividing this figure by the total number of prescriptions written during the period under scrutiny. The aspirational target value given was 90% for each one of the 6R. Additionally, we defined a global quality indicator that measured all the 6R that appeared correctly in the DP. For this quality indicator, the standard value given was 50%.

The classification of ME considered for quality indicators is shown in Table 1.

**Table 1.**
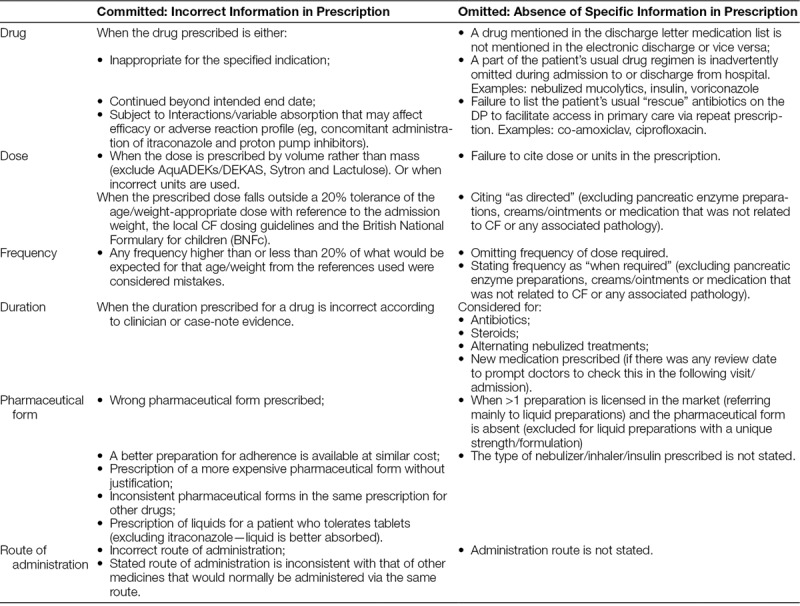
Definition of the Type and Subtype Classification of the Medication/Prescribing Errors

We categorized the severity of the ME depending on the potential pharmacotherapy morbidity, using a scale from 1 to 5 in which describes higher severity while going up the values. Grade 1: ME that would not cause any harm or would be reversible (with no vital signs changes) but monitoring would be required. Grade 2: ME that would cause reversible harm (with no vital signs changes) but modification of treatment would be required. Grade 3: ME that would cause reversible harm and would require additional treatment, prolonged hospital stay. Grade 4: ME causing irreversible harm or disability. Grade 5: ME that would cause the patient’s death.

**Fig. 2.**
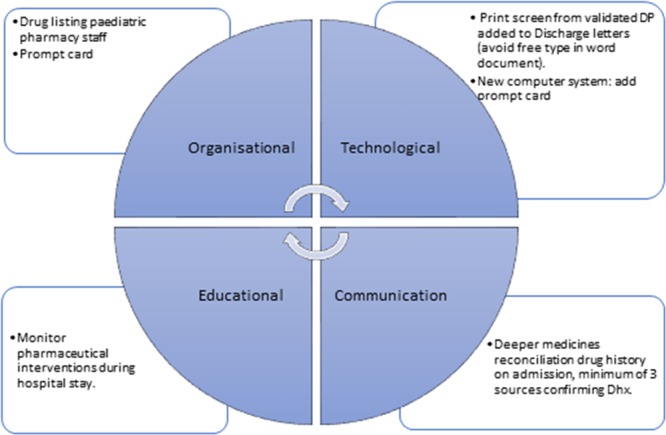
Safety actions and improvement strategies.

Figure 2 represents the strategies to improve DPs and safety actions that the multidisciplinary team agreed to and implemented for CF patients in the prospective phase.

Continuous and categorical variables were analyzed using appropriate statistical tests concerning the type of variable and its distribution. Quantitative data followed a normal distribution (Kolmogorov-Smirnov test applied) and was analyzed by descriptive statistics (ie, mean, SD, and range). We used the Student *t* test when comparing 2 data sets and Analysis of Variance when comparing >2 samples. Chi-square test was applied to compare proportions. We present categorical data as absolute frequencies and relatives (proportions or percentages) with a 95% CI (Table 2).

**Table 2.**
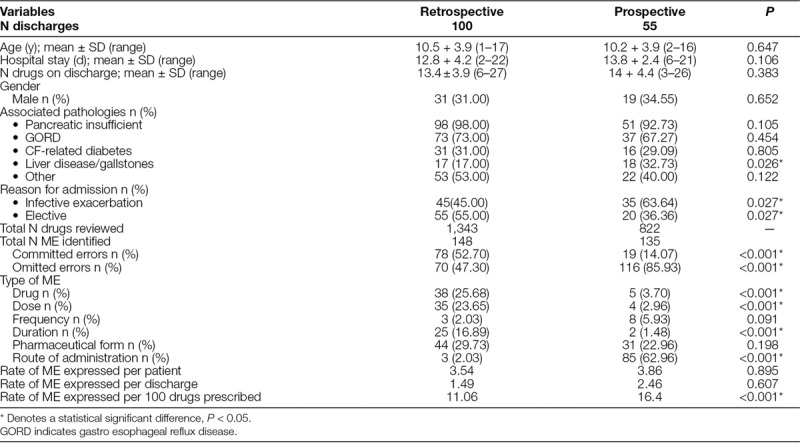
Demographic variables of the discharged patients included in the study and Results of ME.

## RESULTS

We included a total of 42 children, 16 (38%) males and 26 (62%) females, mean age 9.80 years *+* 4.43 (range 1–17) in the retrospective study. The prospective study comprised 35 patients, 14 (40%) males and 21 (60%) females, mean age 10.11 years + 3.89 (range 3–16). The mean number of admissions per patient was 2.43 *+* 2.04 (100 total discharges, range 1–8) in the retrospective phase and 1.60 *+* 0.8 (55 total discharges, range 1–4) in the prospective phase. Table 2 describes the characteristics of the discharged patients included in both phases of the study, type of ME (omitted/committed) with classification according to the 6R, and rates of ME. During medicines reconciliation on admission, a 78% of ME detected corresponded to Grade 1 and 22% of ME caused reversible harm (Grade 2). At discharge, 93% of ME were classified as Grade 1 and 7% as Grade 2. There were no Grade 3 or above ME.

**Fig. 3.**
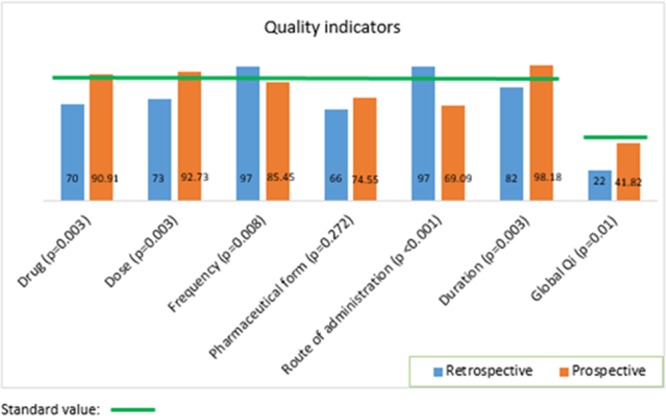
Quality indicator values in both phases of the study with the standard value indicated in green.

Figure 3 shows the values of each quality indicator of the 6R and the global indicator obtained in both phases of the study. Statistical *P*-value when compared quality indicator values obtained in retrospective and prospective studies are also shown.

## DISCUSSION

Previous research has acknowledged that pediatric patients are at risk of encountering ME, including prescribing errors.^16^

A general definition of a prescribing error was developed for pediatrics by Ghaleb et al,^17^ and the following factors were considered prescribing errors: (1) failure to communicate essential information, (2) transcription errors from one prescription to another and the use of drugs, (3) formulations, and (4) inappropriate doses.

Few studies evaluate the incidence of prescribing errors in the CF pediatric population, despite the elevated risk described in the introduction. There have been several UK studies of pharmacist-led interventions, but few involve pharmacist evaluation of CF DPs.

Huynh et al. evaluated the discrepancies of medicines reconciliation in children at the time of hospital admission finding that 45% of the children had at least 1 unintentional medication discrepancy. No single source of information provided all the relevant details of a patient’s medication history. Parents/carers provided the most accurate details of a patient’s medication history in 81% of cases. It is not surprising that children admitted to hospitals are at risk of harm from unintended medication discrepancies at the transition of care from the community to the hospital.^18^

Therefore, children on excessive polytherapy, are vulnerable to potential transitional care ME, especially if there is a low threshold to hospital admissions such as in CF or other complex diseases with associated pathologies.

A possible cause/risk of error is that CF patients on admission for antibiotic IV therapy tend to stop their nebulized antibiotics and in most of the cases patients are expected to restart them after the IV course. However, when the patient is due to be discharged, the information regarding restarting treatment with the nebulized antibiotics and prophylactic antibiotics (if they were on them before admission) can be missed, with the possibility of creating confusion to parents/carers, especially when the burden of responsibility for the child’s CF regimen resides with a specific family member.

Moreover, the pediatric population is notable for wide-ranging weight, physiological, and emotional maturity.^19^ Unfortunately, child-friendly drug formulations remain scarce and often pharmacists must find unlicensed/off license alternatives. Crushing and dispersing tablets is common practice in the pediatric setting, with potential impacts on absorption and adheres, as palatability and can be a problem.

The quality of a prescription for any pediatric patient should be accepted with higher standards to that of a normal prescription. Key among the multiple factors that reduce compliance with CF therapy is the problem of patients receiving conflicting information.^20^ Novel treatments mean the pediatric CF population is ever more vulnerable to ME, and thus, this work is both important and timely.

The professionals involved in prescribing are diverse, for instance: junior doctors (ward prescribing, discharge summaries); senior doctors/consultants (primarily in the outpatient clinic); CF-independent prescribing nurse/pharmacists (clinic letters and homecare prescribing); general practitioners (acute and repeat prescriptions); community pharmacists (prescriptions and over the counter products); and ward pharmacists. This large and varied population of involved professionals may have a different understanding of and training in CF prescribing quality.

Smeulers et al^21^ reviewed the literature to identify evidence-based quality indicators for safe in-hospital medication preparation and administration, and although the current study is based on prescribing indicators, the quality indicators identified by Smeulers were an excellent starting point to develop prescribing specific quality indicators for medication safety. In the current study, we defined the 6R of prescribing, making a further distinction into committed or omitted errors. We established arbitrary aspirational standards for each quality indicator, with targets dependent upon the expectations of the investigator team.

The advantage of the prospective study was that the committed errors of dose and frequency and their potential impact were discussed with the prescribers. Action was taken to rectify them by contacting the parents directly if they had already left the hospital and making sure that the error would not reach the administration stage. (eg, azithromycin would not be given 3 times a day but 3 times a week, which would be quite unlikely as this was part of their regular medication and parents were well aware of their children’s treatment). Also, we encountered some practical obstacles in the pursuit of this work such as weekends discharges written by nonrespiratory specialists (as they are not necessarily aware of the need of restarting medication held during the admission). The addition of pharmaceutical form preferred by the patient in the DP was helpful to ensure there would be continuity of the same form in primary care.

The potential severity of the errors was higher on admission than at discharge, and since the CF department has a fluent communication between the team, together with the culture of sharing mistakes, the tendency of these prescribing errors feels to be minimized as the time is passing since the team is gaining greater experience.

The pediatric pharmacy team is known to the CF department, and this relationship permits closer communication and understanding of the CF patients drug therapy needs. This familiarity is reflected in the way that pharmacists handle DPs: ensuring accuracy of usual patient’s pharmaceutical forms were written down in the DP as well as getting the discharge medication in an optimized timely manner.

Our aspirational target was 90% for each 6R indicator in each DP. Assuming that 90% accuracy target would equate each of the 6R, the global indicator aimed 50% target for the overall quality of prescription in CF children.

We evaluated the quality indicators retrospectively in the first phase of the study. We implemented a new computer system for prescribing at discharge during the final time of the retrospective analysis. This change drove the development of a prescribing prompt card and other strategies to improve prescribing quality prospectively.

The strategies implemented aimed to minimize the error rate at discharge at the same time as ensuring an accurate drug history to serve as a reliable source for future reference.

One of the limitations of the study was that the prospective phase had to be performed in a different prescribing system from the retrospective phase. The new system showed an important procedural error in some prescribing process of the 6R. Each drug had to be manually entered (we did not count minor typographic errors as drug errors); the frequency field was a checkbox type, with no room to edit or expand on specifics of timing, etc; there was no unique database field for route of administration or pharmaceutical form and thus no built-in prompt for prescribers to provide this key information. However, the section to enter the dose and treatment duration of treatment was comparable to the electronic prescribing system used in the retrospective phase, and there was a field to annotate any comments made by pharmacy staff, which were mostly to clarify duration or review dates of treatments (this field was not in the previous system).

The indicators of the drug, dose, and duration showed significant improvement (*P* < 0.05) following the intervention, achieving the quality standard of 90%. The pharmaceutical form indicator showed an improvement from the retrospective phase results but did not achieve the quality standard aimed at 90% (66% versus 74.55% prospectively). By comparison, the frequency indicator deteriorated following the intervention (97% versus. 85.45%, *P* = 0.008), the reasons for which are unclear. There was also a deterioration in the route of administration indicator, largely due to information omitted in the computer system used in the prospective study. The summary quality indicator did not reach the global standard value defined of 50%, although there was an overall improvement (*P* = 0.009), indicative of a modest benefit from the improvement strategies, and a need for further efforts for change.

Having a computer prescribing system that does not prompt users to input complete prescription information challenges prescribers and pharmacists in an overloaded work environment. The current UK healthcare context is marked by significant inpatient bed pressure, with an urgent need to expedite hospital discharge. This pressure lends itself to haste in DP, with junior doctors reliant on the advice of validating ward pharmacists to vouchsafe prescription accuracy. Prescription validation is compromised in that medication reconciliation may not always employ 2 sources of confirmation.

The process described should be embedded as part of a continuous program of quality improvement, to maintain and raise departmental prescribing standards and protect patient safety.

As conclusions in this study, specialist pharmacist review of DPs permits the identification of failures in the quality of prescriptions in children with CF. The implementation of safety actions and improvement strategies designed in this study contributed to an improvement in the prescribing, with an associated reduction in committed errors. The global quality indicator increased significantly with the improvement actions in the prospective phase, although we did achieve the quality standard goal. Continued efforts will drive this indicator to and beyond the target. This improvement will occur through more rigorous implementation of the identified safety strategies, and a rolling program of service evaluation and quality improvement using the methodology outlined in this work. An adequate complement of well-trained highly specialized pharmacists and pharmacy technicians are key to this continued improvement in the quality and safety of DPs in children with CF.

## ACKNOWLEDGMENTS

SQUIRE 2.0 guidelines were used to write the manuscript.
